# Nicotinamide Deteriorates Post-Stroke Immunodepression Following Cerebral Ischemia–Reperfusion Injury in Mice

**DOI:** 10.3390/biomedicines11082145

**Published:** 2023-07-30

**Authors:** Shih-Huang Tai, Liang-Chun Chao, Sheng-Yang Huang, Hsiao-Wen Lin, Ai-Hua Lee, Yi-Yun Chen, E-Jian Lee

**Affiliations:** Neurophysiology Laboratory, Neurosurgical Service, Department of Surgery, National Cheng Kung University Hospital, College of Medicine, National Cheng Kung University, Tainan 70403, Taiwan

**Keywords:** nicotinamide, neuroprotection, immunosuppression, anti-inflammatory, circulating B cells, stroke

## Abstract

(1) Background: Inducing experimental stroke leads to biphasic immune responses, where the early activation of immune functions is followed by severe immunosuppression accompanied by spleen and thymus atrophy. Nicotinamide, a water-soluble B-group vitamin, is a known neuroprotectant against brain ischemia in animal models. We examined the effect of nicotinamide on the central and peripheral immune response in experimental stroke models. (2) Methods: Nicotinamide (500 mg/kg) or saline was intravenously administered to C57BL/6 mice during reperfusion after transiently occluding the middle cerebral artery or after LPS injection. On day 3, the animals were examined for behavioral performance and were then sacrificed to assess brain infarction, blood–brain barrier (BBB) integrity, and the composition of immune cells in the brain, thymus, spleen, and blood using flow cytometry. (3) Results: Nicotinamide reduced brain infarction and microglia/macrophage activation following MCAo (*p* < 0.05). Similarly, in LPS-injected mice, microglia/macrophage activation was decreased upon treatment with nicotinamide (*p* < 0.05), suggesting a direct inhibitory effect of nicotinamide on microglia/macrophage activation. Nicotinamide decreased the infiltration of neutrophils into the brain parenchyma and ameliorated Evans blue leakage (*p* < 0.05), suggesting that a decreased infiltration of neutrophils could, at least partially, be the result of a more integrated BBB structure following nicotinamide treatment. Our studies also revealed that administering nicotinamide led to retarded B-cell maturation in the spleen and subsequently decreased circulating B cells in the thymus and bloodstream (*p* < 0.05). (4) Conclusions: Cumulatively, nicotinamide decreased brain inflammation caused by ischemia–reperfusion injury, which was mediated by a direct anti-inflammatory effect of nicotinamide and an indirect protective effect on BBB integrity. Administering nicotinamide following brain ischemia resulted in a decrease in circulating B cells. This warrants attention with respect to future clinical applications.

## 1. Introduction

The involvement of inflammatory factors in stroke has been widely studied. After cerebral ischemia, the accumulation of inflammatory cells in the brain parenchyma is accompanied by the destruction of the blood–brain barrier (BBB) [1]. The infiltrating immune cells, including neutrophils, macrophages, and lymphocytes, further enhance brain damage by augmenting local inflammatory reactions. Cerebral ischemia is followed by the atrophy of lymphatic organs, as seen in patients and experimental rodent models [2]. Immunodepression is likely beneficial for a reduction in autoreactivity, which results from exposure of the immune system to the central nervous system (CNS)-specific antigens. A previous study demonstrated that early sympathetic system activation causes immunosuppression following brain ischemia [3]. However, a compromised immune system due to stroke tends to result in spontaneous septicemia, pneumonia, and higher mortality rates [3,4].

Nicotinamide (Nico) is a water-soluble B-group vitamin. It is a precursor of nicotinamide adenine dinucleotide (NAD+) and a co-factor in the electron transport chain. Abundant evidence has shown that Nico is a potent neuroprotectant against cerebral ischemia [5], brain trauma [6,7,8], and neurotoxin-induced CNS damage [9,10]. Administering Nico enhances the energy reservoir in the brain by preventing the depletion of NAD+ consumed by nuclear enzyme poly(ADP-ribose) polymerase (PARP), a DNA repair enzyme, thereby maintaining ATP levels in the brain.

In vitro studies have revealed that Nico exhibits multiple inhibitory effects on inflammation and oxidative stress. It inhibits the production of key proinflammatory cytokines, including interleukin 6 (IL-6), IL-1 beta, tumor necrosis factor alpha (TNF-α), and IL-8 [8,11]. Nico also reduces the generation of nitric oxide (NO) and the expression of inducible nitric oxide synthase (iNOS) [12] whilst scavenging free radicals [13]. Additionally, it downregulates the COX-2 expression in macrophages [14] and suppresses the expression of major histocompatibility complex (MHC) class II and intercellular adhesion molecule-1 (ICAM-1) [15,16]. 

Previous research has primarily focused on two aspects of Nico: Its potential to regulate energy metabolism [17,18] and its ability to inhibit programmed cell death [19]. This is because Nico serves as an essential precursor for nicotinamide adenine dinucleotide and acts as an inhibitor for both poly(ADP-ribose) polymerase and sirtuin. We previously demonstrated that Nico effectively attenuated post-ischemic nuclear factor-kappa B activation and exhibited a robust anti-inflammatory action against ischemic stroke [20].

Despite these promising in vitro results, the specific anti-inflammatory effects of Nico in animal stroke models have not been assessed. Our objective in this study was to investigate whether administering Nico could reduce brain inflammation and potentially alter the cellular composition in the peripheral immune system following brain ischemia.

By addressing these gaps in knowledge and evaluating the anti-inflammatory effects of Nico in an animal stroke model, we aimed to establish the potential of Nico as a novel therapeutic agent for ischemic stroke, which could play a significant role in future treatments. Our findings contribute to a deeper understanding of the complex inflammatory processes involved in stroke, paving the way for future therapeutic interventions.

## 2. Materials and Methods

### 2.1. Animals and Grouping

All procedures performed on experimental animals were approved by the Subcommittee on Research Animal Care of the National Cheng Kung University (NCKU) Medical Center (No. 103178). Adult male C57BL/6 mice weighing 20–26 g were supplied by the NCKU Laboratory Animal Center and National Laboratory Animal Center (Tainan, Taiwan). This study was performed in accordance with the relevant guidelines and regulations. Sham-operated mice received identical surgical procedures, excluding vascular opening and filament insertion. Animals were randomly assigned to the nicotinamide treatment group or control group using a computer-based random-order generator. Nicotinamide (Sigma-Aldrich Co., St. Louis, MO, USA) was dissolved in normal saline (0.9% *w*/*v*) for administration. Nicotinamide (500 mg/kg, intravenous injection) or a vehicle (saline) were intravenously delivered to the mice at the onset of reperfusion. There were 9 animals in the control group and 9 animals in the nicotinamide treatment group for the histological analysis and neurobehavioral testing. For the flow cytometry analysis of MCAo-operated animals, there were 7–9 animals in the sham group, 17 animals in the control group, and 22 animals in the nicotinamide treatment group. For the LPS injection model, there were 5 animals in the sham group, 11 animals in the control group, and 9 animals in the nicotinamide treatment group. For the Evans blue leakage experiment, there were 12 animals in the control group and 11 animals in the nicotinamide treatment group.

### 2.2. Transient Middle Cerebral Artery Occlusion (MCAo) Model

C57BL/6 mice anesthetized with 1% halothane in 70% N_2_O/30% O_2_ were subjected to an intra-arterial filament occlusion of the right proximal middle cerebral artery (MCA) for 60 min, as previously described [21,22]. During surgery, the body temperature of the animals was maintained at 37.0 ± 1.0 °C using a rectal probe connected to a thermostatically controlled heating blanket (Harvard Apparatus, South Natick, MA, USA) and a heating lamp. An adequate occlusion was confirmed using laser Doppler flowmetry (LaserFlo BMP2, Vasamedics, St. Paul, MN, USA). The animals were included in the study if they underwent a successful MCA occlusion, which was defined by a 70% or greater drop in cerebral blood flow observed in the laser Doppler flowmetry. The animals were excluded if the insertion of the thread resulted in the perforation of the vessel wall, if the silicon tip of the thread became dislodged during withdrawal, or if the animal died prematurely, preventing the collection of behavioral and histological data. Sham-operated mice received identical surgical procedures, excluding vascular opening and filament insertion. 

### 2.3. Quantitative Measurement of Brain Infarction Volume and Swelling

Behavioral performance was assessed 3 days post-injury. Animals were euthanized under deep anesthesia and perfused with cold Phosphate buffered saline (PBS) through the ascending aorta. Cerebral infarction was determined by staining 40 µm brain tissue cryosections (HM-500O; Microm International GmbH, Walldorf, Germany) with 0.5% Cresyl violet for 4 h at room temperature. Under light microscopy, the areas of neuronal perikarya displaying typical morphological features of ischemic damage were delineated. The infarction volume was measured using a computerized image analyzer (MCID Elite, Imaging Research Inc., St. Catharines, ON, Canada). The infarction volume and brain swelling are expressed as a percentage of the contralateral hemisphere volume, as previously described [23]. In brief, the ipsilateral (right) hemisphere suffered ischemia insults, and the contralateral hemisphere volume was determined using an indirect method. The method involved using the size of the intact contralateral (left) hemisphere to deduct the area of the intact region of the ipsilateral (right) hemisphere to compensate for edema formation in the ipsilateral hemisphere. Ipsilateral brain edema was determined through the summation of the volume increment of the right hemisphere in each brain section relative to the corresponding volume of the left hemisphere. It is expressed as a percentage index relative to the volume of the left hemisphere.

### 2.4. Neurobehavioral Testing and Body Weight Measurements

Body weight measurements and behavioral scales using neurological grading systems were obtained prior to surgery and 3 days after reperfusion by two observers unaware of the treatment protocol, as previously described [24]. The affected forelimb underwent forward and sideways visual placing tests, which were scored as follows: (0) complete immediate placement; (1) incomplete and/or delayed placement (<2 s); and (2) absence of placement. The five categories of motor neurologic findings were scored as follows: (0) no observable deficit; (1) forelimb flexion; (2) forelimb flexion and decreased resistance to lateral push; (3) forelimb flexion, decreased resistance to lateral push and unilateral circling; and (4) forelimb flexion, unable or difficult to ambulate. A grading scale of 0–28 was used, as developed by Clark et al. [25]. The higher the score, the higher the severity of the brain defect caused by the brain injury. The motor performance was examined using a Rota-Rod treadmill (Ugo Basile Biological Research Apparatus, Varese, Italy), which is a sensitive system assessing motor impairments and testing balance and coordination [26,27]. The animals were trained to stay on the rod and were allowed to ambulate until they fell from the rod, or the maximum observation time (300 s) had elapsed. Two different parameters were set in this test. One was an acceleration test (Acc), where mice were placed on the Rota-Rod starting at 2 rpm. This was slowly accelerated to 20 rpm within 5 min. The other test was a fixed speed test (Fix), where the mice were ambulated on the rod, which was rotated at 15 rpm.

### 2.5. Lipopolysaccharide (LPS) Injection

C57BL/6 mice anesthetized with 1% halothane in 70% N_2_O/30% O_2_ were subjected to lipopolysaccharide injection. LPS injection (7.5 mg/kg, intravenous injection, Sigma-Aldrich Co., St. Louis, MO, USA) prior to Nicotinamide (500 mg/kg, intravenous injection) or a vehicle (saline) treatment. The animals were euthanized after 3 days. 

### 2.6. Isolation of Cells from the Brain, Spleen, Thymus, and Blood

After a saline perfusion, the brain was divided into ischemic right and nonischemic left hemispheres. These were then chemically dissociated with papain (Sigma-Aldrich Co., St. Louis, MO, UAS), mechanically triturated, and passed through a 70 μm mesh. CNS mononuclear cells were isolated using a 30%, 37%, and 70% Percoll gradient with centrifugation at 500× *g* for 40 min, as previously described [28]. Cells in the spleen and thymus were flushed out with PBS using needles and syringes. Single-cell suspensions were prepared through a 70 μm nylon mesh. Cardiac blood was collected in a heparin-rinsed needle/syringe. Red blood cells were lysed with an Ammonium-Chloride-Potassium (ACK) lysing buffer (0.15 mol/L NH4Cl, 10 mmol/L KHCO_3_, and 0.1 mmol/L EDTA) for 6 min followed by PBS washing. The cells were resuspended in a FACS staining buffer (0.5% bovine serum albumin and 0.02% sodium azide in PBS) and counted using a hemocytometer before antibody staining.

### 2.7. Flow Cytometry

Three-color fluorescence flow cytometric analyses were performed to determine the phenotypes of the brain and blood mononuclear cells. Cells were blocked with an FcR antibody (553141; BD Biosciences, San Jose, CA, USA) for 30 min and stained with a combination of the following antibodies for 30 min at room temperature: CD11b (553310 and 553311), LY6G (551460), CD45 (553081), CD11c (553802), IgM (553437), and IgD (558597, purchased from BD Biosciences, San Jose, CA, USA), CD19 (115506) and CD23 (101614, purchased from Biolegend, San Diego, CA, USA), and CD3 (HM3401, purchased from Caltag). After antibody staining, cells were sorted using a FACSCalibur system (BD Biosciences, San Jose, CA, USA). Forward and side scatters were used to identify lymphocytes. Dead cells were gated out using propidium iodide discrimination. Data were analyzed using Cell Quest. For each experiment, cells were stained with appropriate isotype control antibodies to establish background staining and set quadrants to calculate the percentage of positive cells. The microglia/macrophage percentage was determined by subtracting the percentage of CD11b+/LY6G+ (neutrophils) from the percentage of CD45+/CD11b+ [28,29]. The percentage of the activated form of microglia/macrophage was determined as follows:

(1) $$\frac{\left(CD45^{hi}/CD11b^{hi}-CD11b+/LY6G+\right)}{(CD45^{hi}/CD11b^{hi}-CD11b+/LY6G+)+CD45^{low}/CD11b^{low}}\;\;\;\;\;\;\%$$

### 2.8. Measurement of Evans Blue Leakage 

Evans blue (Sigma-Aldrich Co., St. Louis, MO, USA) extravasation was quantified as previously reported [30]. Briefly, 0.1 mL of 1% Evans blue dissolved in 0.9% saline was injected via the right carotid vein during reperfusion following MCAo. Animals were anesthetized with 1% halothane in 70% N_2_O/30% O_2_ 24 h after the reperfusion and underwent transcardiac perfusion with saline until the blood was entirely removed. The brain was separated into ischemic and nonischemic hemispheres, weighed, and frozen at −80 °C until analyzed. Each hemisphere was homogenized in 50% trichloroacetic acid (*w*/*v*) and centrifuged at 18,000× *g* for 10 min at room temperature. The supernatant was diluted in 100% ethanol, and the fluorescence of the Evans blue was measured using a plate reader (Stat Fax 2100, Awareness Technology, Inc., Palm City, FL, USA) at 620/680 nm (excitation/emission, respectively). 

### 2.9. Statistical Analysis

Data were analyzed using a one-way ANOVA followed by Tukey’s post hoc test or a Student’s *t*-test to detect significant differences between the means, with *p* < 0.05 indicating significance. Neurobehavioral scores were analyzed using the Mann–Whitney U test. Data are presented as the mean ± standard deviation (SD). The analysis of data was performed using SPSS Statistics for Windows version 17.0 (SPSS Inc., Chicago, IL, USA).

## 3. Results

### 3.1. Nicotinamide Reduces Brain Infarction and Improves Neurobehavior Outcomes Following Ischemia–Reperfusion Injury

**Figure 1:**
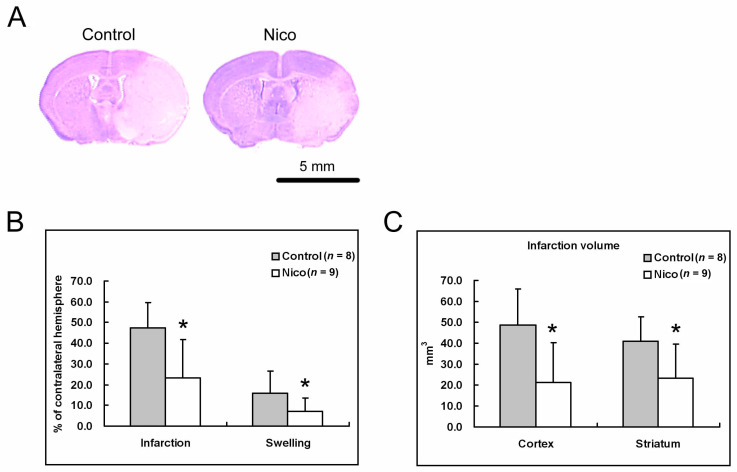
Nicotinamide reduced brain infarction and swelling 3 days after MCAo. (**A**) Representative Nissl-stained sections from control and Nico-treated mice. (**B**) Nicotinamide decreased brain infarction and swelling. (**C**) Nicotinamide decreased both cortical and striatal infarctions. Data presented as mean ± SD; *n* = 9; * *p* < 0.05, Student’s *t*-test.

The mice were subjected to MCA occlusion and randomly assigned to Nico-treated or control groups. Treatment with nicotinamide did not significantly alter the local cortical blood perfusion or core temperature during the surgery. Brains of the vehicle or Nico-treated MCAo mice were cryosectioned and stained with Cresyl violet (Nissl staining) to calculate brain infarction and swelling (Figure 1A). The damaged cells exhibited shrunk nuclei and cell bodies. Treatment with nicotinamide resulted in approximately a 50.8% reduction in infarction volume (47.3 ± 11.3% for the control mice versus 23.3 ± 18.3% for the Nico-treated mice; *p* < 0.05; Figure 1B). Administering nicotinamide also reduced brain swelling by 54.0% (15.7 ± 10.0% for the control mice versus 7.2 ± 6.2% for the Nico-treated mice; *p* < 0.05; Figure 1B). The decrease in infarction was prominent in both the cortex and striatum, the volume decreasing by 56.6% (49.5 ± 16.6% for the control mice versus 21.5 ± 19.0% for the Nico-treated mice; *p* < 0.05; Figure 1C) and 42.5% (40.4 ± 11.0% for the control mice versus 23.2 ± 16.3% for the Nico-treated mice; *p* < 0.05), respectively. Examining behavioral performance using a 28-point sensorimotor score and the Rota-Rod test showed that nicotinamide significantly improved the behavioral performance of the animals and reduced weight loss (*p* < 0.05; Table 1).

### 3.2. Nicotinamide Decreases Brain Inflammation Following Ischemia–Reperfusion Injury

**Figure 2:**
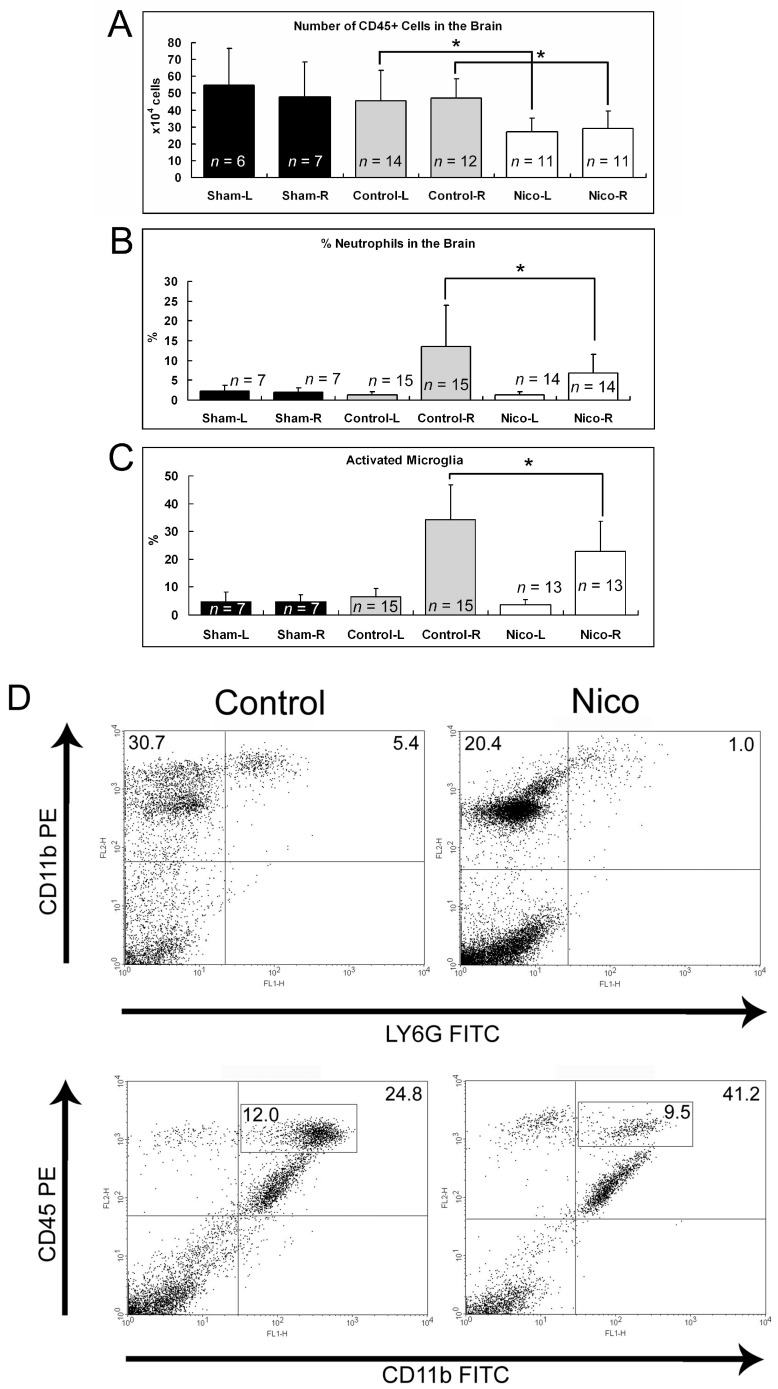
Effects of nicotinamide on brain inflammation following MCAo after 3 days. (**A**) Nicotinamide decreased the number of immune cells in both ischemic and nonischemic hemispheres. (**B**) Nicotinamide decreased the percentage of infiltrated neutrophils in the ischemic hemisphere. (**C**) Nicotinamide reduced the percentage of activated microglia/macrophages in the ischemic hemisphere. (**D**) Representative flow cytometry dot plots of neutrophils and activated microglia in the ischemic hemisphere. Data presented as mean ± SD; * *p* < 0.05; one-way ANOVA followed by Tukey’s post hoc test.

The effect of nicotinamide on brain inflammation following brain ischemia was assessed in ischemic and nonischemic hemispheres 3 days post-MCAo. The nicotinamide treatment significantly reduced the number of immune cells (CD45+) in both ischemic and nonischemic hemispheres by approximately 37.9% and 40.3%, respectively (47.0 ± 11.7 and 29.2 ± 10.3 × 10^4^ cells in the right/ischemic hemispheres of the control and Nico-treated mice, respectively, and 45.4 ± 18.2 and 27.1 ± 7.9 × 10^4^ cells in the left/nonischemic hemispheres of the control and Nico-treated mice, respectively; *p* < 0.05; Figure 2A). The nicotinamide treatment significantly inhibited the infiltration of neutrophils into the ischemic hemisphere by approximately 48.9% (13.5 ± 10.4% for the control mice versus 6.9 ± 4.6% for the Nico-treated mice; *p* < 0.05; Figure 2B,D). In contrast, T and B cell infiltration into the ischemic hemisphere was minimal and was unchanged after the nicotinamide treatment (Table S3A). The activated form of the microglia/macrophage showed an elevated expression of CD45 and CD11b [28,31]. Our results show that administering nicotinamide decreased microglia/macrophage activation in the ischemic hemisphere by 33.5% compared with the control (34.3 ± 12.5% for the control mice versus 22.8 ± 10.9% for the Nico-treated mice; *p* < 0.05; Figure 2C,D).

### 3.3. Nicotinamide Suppresses Microglial Activation Induced by Lipopolysaccharide (LPS)

**Figure 3:**
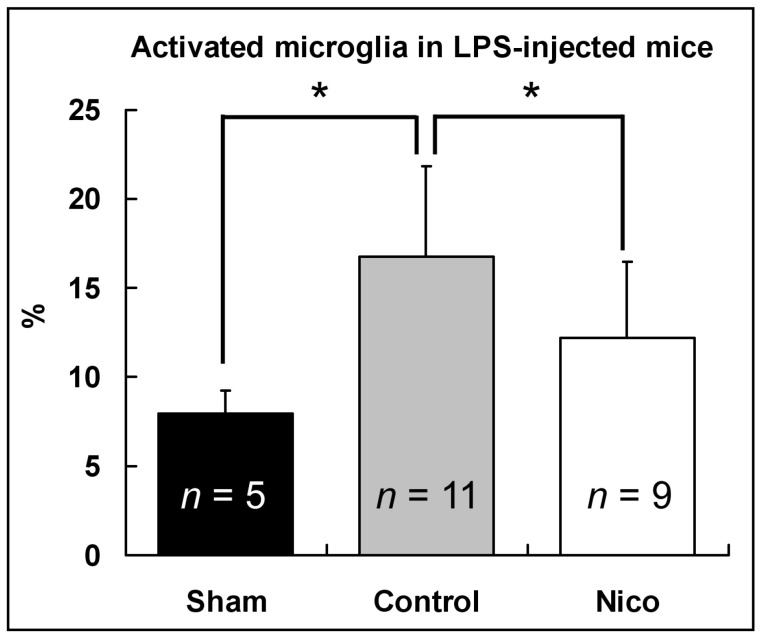
Nicotinamide reduced microglia/macrophage activation following LPS injection at 3 days. Data presented as mean ± SD; * *p* < 0.05; one-way ANOVA followed by Tukey’s post hoc test.

To determine whether the inhibitory effect of nicotinamide on microglia/macrophage activation was solely a result of reduced brain damage, we assessed the effect of nicotinamide in a pure inflammation model induced by an intravenous injection of LPS. The LPS injection enhanced microglia/macrophage activation 2.1-fold (*p* < 0.05; Figure 3), and nicotinamide suppressed the activation by 32.8% (7.9 ± 1.3% for the sham mice versus 16.8 ± 5.0% for the control mice versus 11.3 ± 3.5% for the Nico-treated mice; *p* < 0.05). The results demonstrate that nicotinamide plays a direct role in inhibiting microglial activation.

### 3.4. Nicotinamide Ameliorates BBB Leakage Following MCAo

**Figure 4:**
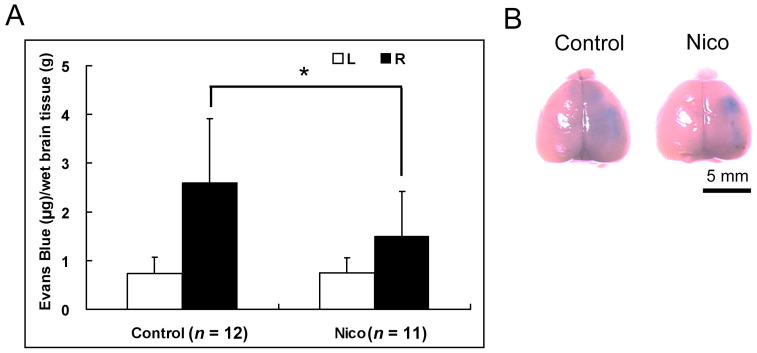
Nicotinamide decreased Evans blue leakage following MCAo on day 1. (**A**) Nicotinamide decreased Evans blue leakage in the ischemic hemisphere following MCAo. Data presented as mean ± SD; * *p* < 0.05; Student’s *t*-test. (**B**) Representative images of brains from Evans blue-injected MCAo control or Nico-treated mice.

We further evaluated the permeability of the BBB, which is a physical and metabolic barrier between the CNS and systemic circulation. The perturbation or disruption of the BBB could result in elevated transmigration of immune cells [32,33]. Administering nicotinamide reduced BBB leakage in the ischemic hemisphere by 42.5% (2.59 ± 1.33 μg/g for the control mice versus 1.49 ± 0.93 μg/g for the Nico-treated mice; *p* < 0.05; Figure 4). The results demonstrate that in Nico-treated mice, the reduced neutrophil infiltration was, at least in part, mediated by reduced BBB leakage.

### 3.5. Nicotinamide Reduces Circulating B Cells in the Thymus and Blood Following MCAo

**Figure 5:**
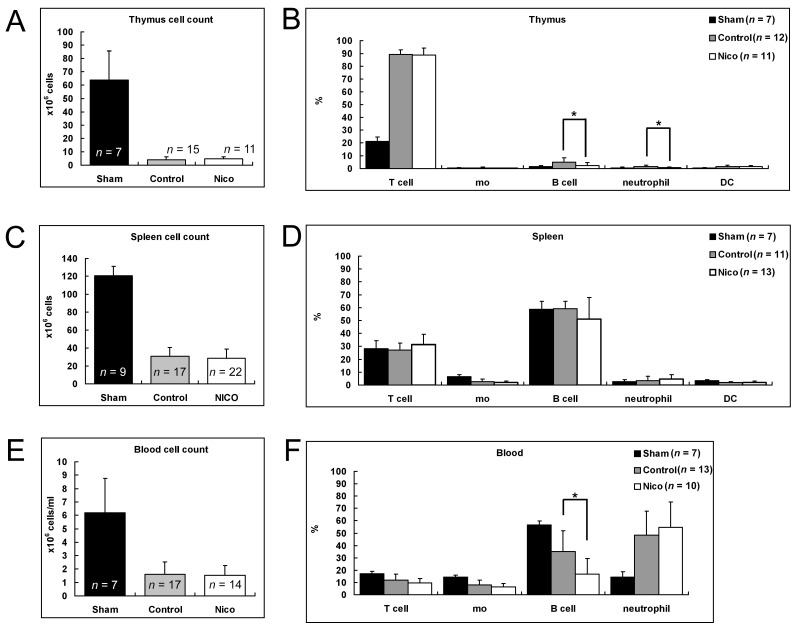
Effects of nicotinamide on peripheral immune organs following MCAo after 3 days. Administering nicotinamide did not change the cell numbers in the thymus (**A**), spleen (**C**), and blood (**E**). It reduced the proportion of B cells in the thymus (**B**) and blood (**F**), but not in the spleen (**D**). Data presented as mean ± SD; * *p* < 0.05; one-way ANOVA followed by Tukey’s post hoc test.

Extensive studies have shown that brain ischemia leads to the atrophy of peripheral immune organs [34]. We examined the effects of nicotinamide on peripheral immune organs following MCAo. The number of immune cells was significantly reduced in the thymus, spleen, and blood. The nicotinamide treatment did not alter the cell number in these organs (*p* > 0.05; Figure 5A,C,E). Interestingly, nicotinamide treatment altered the composition of cells in the thymus and blood. Administering nicotinamide decreased circulating B cells in the thymus by 50.9% (5.1 ± 3.3% for the control mice versus 2.5 ± 2.1% for the Nico-treated mice; *p* < 0.05; Figure 5B) and those in the blood by 52.0% (35.2 ± 16.7 for the control mice versus 16.9 ± 12.5% for the Nico-treated mice; *p* < 0.05; Figure 5F). The nicotinamide treatment did not change the composition of other cell types in the thymus and blood. None of the specific phenotypes of the cells examined in the spleen changed with the nicotinamide treatment (Figure 5D).

### 3.6. Nicotinamide Retards B Cell Maturation in the Spleen Following MCAo

**Figure 6:**
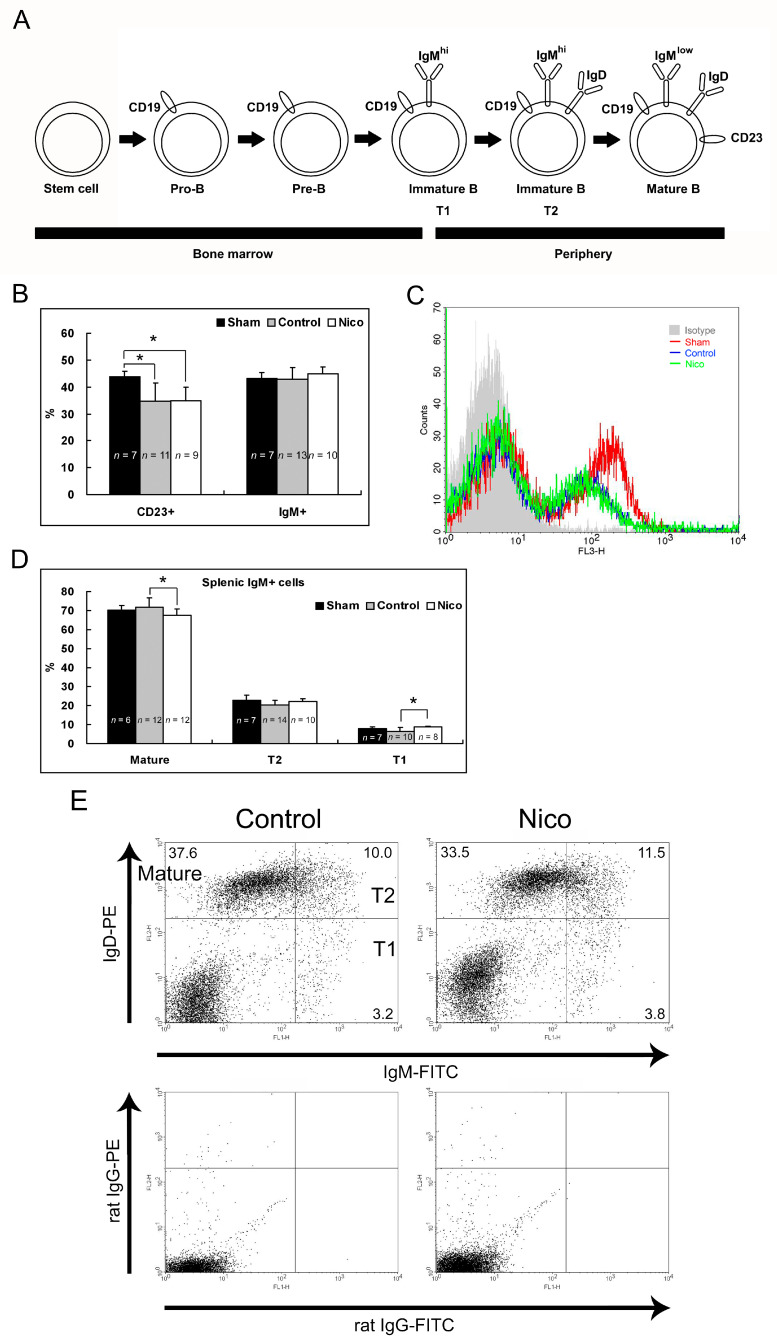
Administering nicotinamide arrested B cell maturation in the T1 phase in the spleen 3 days following MCAo. (**A**) Schematic illustration of the phenotypic markers expressed in B cells at different maturation stages. (**B**) MCAo reduced the percentage of CD23+ but not IgM+ cells in the spleen. (**C**) Representative histogram of CD23 expression. (**D**) Nicotinamide increased the percentage of T1 in the spleen, subsequently decreasing the percentage of mature IgM-expressing cells. (**E**) Representative flow cytometry dot plots of mature B cells and immature B cells in the spleen. Data presented as mean ± SD; * *p* < 0.05; one-way ANOVA followed by Tukey’s post hoc test.

To understand whether the reduced number of circulating B cells after the nicotinamide treatment in the MCAo model was a result of retarded B cell maturation, we analyzed the CD23, IgM, and IgD expression in the spleens of the sham-operated, control, and Nico-treated mice 3 days following MCAo (Figure 6A). Ischemia–reperfusion brain injury led to a significant reduction (20.7%) in the percentage of CD23+ cells in the spleen (43.8 ± 2.1% for the sham mice versus 34.8 ± 6.7% for the control mice; *p* < 0.05), but not of IgM+ cells (*p* > 0.05; Figure 6B,C). The nicotinamide treatment failed to decrease the percentage of CD23+ or IgM+ cells in the spleen (*p* > 0.05). Mature and transitional B cells express different levels of IgM and IgD proteins on the surface: Mature (IgM^low^/IgD+), transitional 1 (T1; IgM^hi^/IgD−) and transitional 2 (T2; IgM^hi^/IgD+) B cells could be determined [35]. We observed that in the total IgM-expressing cells, the nicotinamide treatment increased the proportion of T1 cells by 36.5% (6.4 ± 2.1% for the control mice versus 8.7 ± 0.6% for the Nico-treated mice; *p* < 0.05), resulting in a small but significant reduction in the proportion of mature B cells of 6.1% (71.7 ± 4.9% for the control mice versus 67.4 ± 3.3% for the Nico-treated mice; *p* < 0.05; Figure 6D,E). In contrast, the proportion of T2 was not altered upon treatment with nicotinamide (*p* > 0.05).

## 4. Discussion

Our results confirm that nicotinamide at 500 mg/kg is protective against ischemia–reperfusion injury in the brain as it significantly reduced brain infarctions and improved neurological functions. An assessment of the Nissl-stained sections showed that nicotinamide reduced brain swelling, which was consistent with the improved BBB integrity measured using Evans blue leakage. Data from these studies showed that Nico had a direct inhibitory effect on microglia/macrophage activation. Together with its function in ameliorating BBB integrity, nicotinamide showed an overall suppressive effect on stroke-induced brain inflammation. Cumulatively, in addition to the heightened energy reservoir of NAD+ directly supplemented by nicotinamide, the neuroprotective effects of nicotinamide could be multi-faceted. 

Similar to the flow cytometry analyses by Stevens et al. [31], neutrophil infiltration and microglial/macrophage activation were significantly enhanced in the ischemic hemisphere compared with the contralateral nonischemic hemisphere at day 3 post-MCAo [31]. As neutrophils (LY6G+) also express high levels of CD11b, we subtracted the LY6G+ cells from the CD45^hi^/CD11b^hi^ population to calculate microglia/macrophage activation. This may have been the reason why we observed a lower percentage of activated microglia/macrophages than Stevens et al. did. The T cells were at low levels in the sham-operated animals. The level remained low in the ischemic hemisphere on day 3 following MCAo. In contrast, the research of Stevens et al. showed a high percentage of T-cell infiltration. However, discrepant results were observed in their flow cytometry and immunohistochemistry studies. Three days after MCAo, they showed that approximately 3.5 cells per field of view were T cells and 15 cells per field of view were neutrophils (approximately a 4-fold difference), whereas the flow cytometry analyses of neutrophils and T cells were at similar levels (approximately 12% and 11% of CD45+ cells, respectively).

The anti-inflammatory effects of nicotinamide have been extensively studied on a cellular level [11,12,15,16,36]. Our results demonstrated that nicotinamide could suppress microglia/macrophage activation in a pure inflammation model induced with endotoxins as well as in an experimental stroke model. The results from the endotoxin-injected mice further justified the conclusion that the inhibition of microglia/macrophage activation by nicotinamide in MCAo mice was not merely a result of reduced brain infarction.

Nicotinamide inhibits the activation of B cells induced by multiple ligands, including anti-IgM, anti-CD40, and LPS [37]. It also prevents the activation of ERK pathways upon stimulation of B-cell receptors. This subsequently inhibits the expression of cyclin D2, which is a downstream product of activated ERK pathways [37,38]. These results suggest that nicotinamide is involved in regulating B-cell proliferation. Our results showed that nicotinamide modulated B-cell maturation after strokes. Cumulatively, our data and those of others suggest that nicotinamide has an inhibitory role in B-cell activation, proliferation, and maturation.

Although nicotinamide protects the brain against ischemic injury, its effect on the peripheral immune system reduces circulating B cells. Immunosuppression after a stroke is seen as a protective mechanism to decrease autoreactive responses against CNS-derived antigens leaking through a disrupted BBB. This is supported by evidence that mice lacking T and B cells had decreased lesion sizes and reduced brain inflammation after brain ischemia compared with wild-type mice [39]. Mice deficient in B cells exhibited similar brain injuries, cerebral inflammation, and neurological scores as wild-type mice following strokes, suggesting that B cells do not contribute to stroke-induced neurological damage [40]. Therefore, the beneficial effects of nicotinamide on the brain following a stroke are not likely to be mediated by B cells. 

A limitation of this study was that only male mice were used. A previous study reported that different genders demonstrated different protective effects of neuroprotectants after ischemic strokes. This was thought to be caused by estrogen [41]. Further research on this issue is required.

## 5. Conclusions

Our study demonstrates that nicotinamide suppresses central and peripheral immune responses following brain ischemia. Administering nicotinamide reduced microglia/macrophage activation, ameliorated BBB dysfunction, decreased brain infarction, and had an inhibitory role in B-cell activation, proliferation, and maturation. Although nicotinamide ameliorated neuroprotection by decreasing BBB damage and neuroinflammation, it is worth noting that the immune system is compromised post-stroke, and nicotinamide may further exacerbate this. A compromised immune system increases the susceptibility of patients to systemic infections after strokes. In the future, the clinical use of nicotinamide in patients after ischemic strokes should be accompanied by increased attention to the risk of infections. However, nicotinamide appears to be a promising candidate for clinical use as an anti-inflammatory and immune-modulating therapeutic agent.

## Figures and Tables

**Table 1:** Nicotinamide improves sensorimotor behavioral scores after cerebral ischemia–reperfusion.

		Neurological Behavioral Score		
	Weight Loss (g)	28-Point Clinical Scale	Rota-Rod (s)	
			Fixed Speed	Accelerated Speed
Vehicle (*n* = 9)	6.7 ± 0.8	17.1 (10.47~23.73)	107.6 ± 52.8	78.2 ± 37.6
Nicotinamide (*n* = 9)	6.0 ± 1.0 *	11.9 (5.21~18.59) *	192.7 ± 78.9 *	187.8 ± 87.4 *

Weight data and Rota-Rod results expressed as mean ± S.D. Neurological behavioral scores are expressed as median (95% CI). Intravenous injection of nicotinamide (500 mg/kg) immediately after reperfusion significantly ameliorated weight loss and improved sensorimotor neurological scores and Rota-Rod running tests compared with vehicle-injected control values. * *p* < 0.05 versus vehicle.

## Data Availability

The data that support the findings of this study are available from the corresponding author upon reasonable request.

## Supplementary Materials

The following supporting information can be downloaded at: https://www.mdpi.com/article/10.3390/biomedicines11082145/s1, Table S1: Blood flow and body temperature; Table S2: Cell counts in MCAo mice; Table S3: Immune cells in thymus, spleen, blood, and brain; Table S4: Immune cells in LPS-stimulated brain; Table S5: CD23, IgM, and IgD expression in the spleen.
