# Supplementary material for: Nicotinamide Deteriorates Post-Stroke Immunodepression Following Cerebral Ischemia–Reperfusion Injury in Mice
Source: Biomedicines. 2023 Jul 30;11(8):2145. doi: 10.3390/biomedicines11082145 (PMC10452067; doi:10.3390/biomedicines11082145)
Supplement: Supplementary file 1 [file biomedicines-11-02145-s001.zip › Table S5 CD23, IgM, and IgD expression in the spleens.pdf]

Supplemental Table

					IgM+ (100%)		
	**Cell count (x10^6)**	**%PI+**	**%CD23+**	**%IgM+**	**%Mature (IgM^{lo}/IgD+)**	**%T2 (IgM^{hi}/IgD+)**	**%T1 (IgM^{hi}/IgD-)**
**Sham**	121.9±14.2	7.2±0.8	43.4±2.6	43.1±1.5	70.7±2.5	21.8±2.4	7.5±0.7
**Vehicle, MCAo**	25.5±9.0#	14.7±5.9#	36.3±7.4	42.8±4.5	71.7±4.9	20.4±2.4	6.4±2.1
**NAm, MCAo**	27.7±10.2#	12.7±3.9#	35.0±4.9#	44.9±2.5	67.4±3.3*	21.9±1.7	8.7±0.6*#

Table S5. CD23, IgM, and IgD expression in the spleens of sham-operated, control, and Nico-treated mice 3 days after MCAo. Data are represented as mean±SD (sham, n=4; Veh, Nam, n=10-13). $^{\#}p$ <0.05 compared with sham-operated mice by one-way ANOVA with Tukey's post-hoc test. *p <0.05 compared with vehicle-treated MCAo mice.
