# Supplementary material for: Nicotinamide Deteriorates Post-Stroke Immunodepression Following Cerebral Ischemia–Reperfusion Injury in Mice
Source: Biomedicines. 2023 Jul 30;11(8):2145. doi: 10.3390/biomedicines11082145 (PMC10452067; doi:10.3390/biomedicines11082145)
Supplement: Supplementary file 1 [file biomedicines-11-02145-s001.zip › Table S1 Blood flow and body temperature.pdf]

Supplemental Table

	Pre-occlusion	MCAo	Before reperfusion	After reperfusion	After drug administration
**LCBF of ischemic core (%)**					
Vehicle	100	9.0 ± 0.8	9.2 ± 1.0	44.5 ± 3.9	46.4 ± 4.0
Nicotinamide	100	8.4 ± 0	8.8 ± 1.3	45.6 ± 3.2	47.9 ± 4.2
**LCBF of ischemic penumbra (%)**					
Vehicle	100	34.6 ± 0.7	34.8 ± 1.5	93.8 ± 5.8	97.6 ± 7.1
Nicotinamide	100	34.4 ± 0.7	34.6 ± 0.9	91.2 ± 4.5	93.4 ± 5.4
**LCBF of contralateral hemisphere (%)**					
Vehicle	100	96.8 ± 3.8	97.1 ± 7.0	99.6 ± 4.4	102.6 ± 4.3
Nicotinamide	100	97.7 ± 4.7	98.8 ± 4.3	99.8 ± 4.3	103.5 ± 3.6
**Core temperature (°C)**					
Vehicle	37.0 ± 0.1	36.6 ± 0.2	35.5 ± 0.4	35.4 ± 0.3	35.4 ± 0.3
Nicotinamide	36.9 ± 0.1	36.7 ± 0.2	35.6 ± 0.4	35.5 ± 0.4	35.5 ± 0.4

Table S1. Local cortical blood perfusion (LCBF) and core temperature obtained prior to (pre-occlusion), immediately and 60 mins after middle cerebral artery occlusion (MCAo), at the onset of reperfusion and after drug administration in animals subjected to MCAo with treatment of nicotinamide or vehicle (normal saline). Data are presented as the mean ± standard deviation. LCBF and core temperature data were normal and did not differ significantly at various time intervals between vehicle- and nicotinamide-treated animals (n=17 per group).
