# Supplementary material for: Nicotinamide Deteriorates Post-Stroke Immunodepression Following Cerebral Ischemia–Reperfusion Injury in Mice
Source: Biomedicines. 2023 Jul 30;11(8):2145. doi: 10.3390/biomedicines11082145 (PMC10452067; doi:10.3390/biomedicines11082145)
Supplement: Supplementary file 1 [file biomedicines-11-02145-s001.zip › Table S2 Cell counts in MCAO mice.pdf]

Supplemental Table

	Thymus weight (mg)	Spleen weight (mg)	Thymus cell count ($x10^6$)	Spleen cell count ($x10^6$)	Blood cell count ($x10^6$/ml)	Number of CD45+ cells in left hemisphere ($x10^4$)	Number of CD45+ cells in right hemisphere ($x10^4$)
**Sham**	36.1±5.6	82.9±7.6	63.9±21.8	121.4±23.1	6.2±2.6	54.9±21.5	47.9±20.7
**Vehicle, MCAo**	11.8±2.2$^{\#}$	50.6±11.2$^{\#}$	4.1±2.3$^{\#}$	48.5±20.3$^{\#}$	1.6±0.9$^{\#}$	45.4±18.2	47.0±11.7
**NAm, MCAo**	12.8±3.2$^{\#}$	47.7±11.8$^{\#}$	4.8±1.6$^{\#}$	28.9±11.4$^{\#}$*	1.6±0.7$^{\#}$	27.1±7.9$^{\#}$*	29.2±10.3$^{\#}$*

Table S2. Cell counts in sham operated or MCAo mice with vehicle or nicotinamide treatment for 3 days. Data are represented as mean±SD (sham, n=7; Veh, Nam, n=10-17). $^{\#}p$ <0.05 compared with sham-operated mice by one-way ANOVA with Tukey's post-hoc test. *p <0.05 compared with vehicle-treated MCAo mice by one-way ANOVA with Tukey's post-hoc test.
