# Supplementary material for: Nicotinamide Deteriorates Post-Stroke Immunodepression Following Cerebral Ischemia–Reperfusion Injury in Mice
Source: Biomedicines. 2023 Jul 30;11(8):2145. doi: 10.3390/biomedicines11082145 (PMC10452067; doi:10.3390/biomedicines11082145)
Supplement: Supplementary file 1 [file biomedicines-11-02145-s001.zip › Table S3 Immune cells in thymus, spleen, blood and brain.pdf]

Supplemental Table

A	Left hemisphere					Right hemisphere				
**(%)**	**T cells**	**Microglia /MO**	**B cells**	**Neutrophils**	**Activated microglia**	**T cells**	**Microglia /MO**	**B cells**	**Neutrophils**	**Activated microglia**
**Sham**	1.1±0.3	89.0±4.8	0.7±0.5	2.3±1.5	4.8±3.4	1.8±0.9	89.5±3.8	0.8±0.5	1.9±1.2	4.8±2.3
**Vehicle, MCAo**	0.4±0.3	94.6±2.8	1.1±0.7	1.3±0.8	6.4±3.1	2.0±1.6†	80.3±11.2$^{\#†}$	2.3±1.3$^{\#†}$	13.5±10.4$^{\#†}$	34.3±12.5$^{\#†}$
**NAm, MCAo**	0.4±0.2	94.1±2.6	0.8±0.6	1.4±0.8	3.7±1.8	1.5±1.0	85.2±6.7†	2.0±1.6	6.9±4.6*†	22.8±10.9$^{\#*†}$

B	Thymus					Spleen				
**(%)**	**T cells**	**MO**	**B cells**	**Neutrophils**	**DC**	**T cells**	**MO**	**B cells**	**Neutrophils**	**DC**
**Sham**	21.3±3.5	0.4±0.4	1.7±0.5	0.5±0.7	0.5±0.4	28.1±6.3	6.5±1.4	58.6±6.1	2.8±1.3	3.5±0.9
**Vehicle, MCAo**	89.3±3.2$^{\#}$	0.5±0.7	5.1±3.3$^{\#}$	1.6±1.1$^{\#}$	1.6±0.9$^{\#}$	27.2±5.1	2.6±1.9$^{\#}$	59.4±5.6	3.5±3.5	2.0±0.8
**NAm, MCAo**	88.7±5.7$^{\#}$	0.2±0.3	2.5±2.1*	0.7±0.5*	1.4±0.7	31.4±7.9	1.7±1.5$^{\#}$	51.2±16.6	4.7±3.3	1.9±1.0

C	Blood			
**(%)**	**T cells**	**MO**	**B cells**	**Neutrophils**
**Sham**	17.0±2.1	14.3±1.7	56.7±3.2	14.5±4.3
**Vehicle, MCAo**	11.9±4.9$^{\#}$	8.0±3.9$^{\#}$	35.2±16.7$^{\#}$	48.4±19.5$^{\#}$
**NAm, MCAo**	9.5±3.7$^{\#}$	6.2±2.9$^{\#}$	16.9±12.5$^{\#*}$	54.4±21.0$^{\#}$

Table S3. Proportion of immune cells in thymus, spleen, blood and brain in sham operated or MCAo mice with vehicle or nicotinamide treatment. Data are represented as mean±SD (sham, n=7; Veh, Nam, n=8-16) #p<0.05 compared with sham-operated mice in the same hemisphere by one-way ANOVA with Tukey's post-hoc test. *p<0.05 compared with vehicle-treated MCAo mice in the same hemisphere by one-way ANOVA with Tukey's post-hoc test. † p<0.05 compared with the respective left hemisphere by one-way ANOVA with Tukey's post-hoc test
