# Supplementary material for: Nicotinamide Deteriorates Post-Stroke Immunodepression Following Cerebral Ischemia–Reperfusion Injury in Mice
Source: Biomedicines. 2023 Jul 30;11(8):2145. doi: 10.3390/biomedicines11082145 (PMC10452067; doi:10.3390/biomedicines11082145)
Supplement: Supplementary file 1 [file biomedicines-11-02145-s001.zip › Table S4 Immune cells in LPS brain.pdf]

Supplemental Table

(%)	Brain				
	**T cells**	**Microglia /MO**	**B cells**	**Neutrophils**	**Activated microglia**
**Sham**	1.0±0.3	83.8±6.1	0.5±0.1	1.4±0.7	7.9±1.3
**LPS + Vehicle**	1.2±0.4	76.8±5.6	0.8±0.6	2.4±1.0	16.8±5.0
**LPS + Nicotinamide**	0.9±0.6	87.3±5.1*	0.4±0.3	2.4±1.1	12.2±4.3*

Table S4. Proportion of immune cells in brain in sham, LPS plus vehicle or nicotinamide mice for 3 days.

Data are presented as mean±SD (Sham, n=6; Control, Nico, n=9-11). * p <0.05 compared with LPS+vehicle.
